# Amyloid myopathy: expanding the clinical spectrum of transthyretin amyloidosis—case report and literature review

**DOI:** 10.1007/s12350-022-02990-x

**Published:** 2022-05-17

**Authors:** Maria Ungericht, Julia Wanschitz, Alexander S. Kroiss, Christoph Röcken, Thomas Schuetz, Moritz Messner, Marc-Michael Zaruba, Wolfgang N. Loescher, Gerhard Poelzl

**Affiliations:** 1 Department of Internal Medicine III, Cardiology & Angiology, Medical University of Innsbruck, Anichstraße 35, 6020 Innsbruck, Austria; 2 Department of Neurology, Medical University of Innsbruck, Innsbruck, Austria; 3 Department of Nuclear Medicine, Medical University of Innsbruck, Innsbruck, Austria; 4 Department of Pathology, Christian-Albrechts-University, Kiel, Germany

**Keywords:** ATTR amyloid myopathy, ^99m^Tc-DPD scintigraphy, myopathy, neuromyopathy, cardiac ATTR amyloidosis, transthyretin, ATTR

## Abstract

We identified two patients with transthyretin (ATTR) amyloid myopathy (one ATTR variant amyloidosis, ATTRv; one wild-type ATTR amyloidosis, ATTRwt). Myopathy was the initial manifestation in ATTRwt, whereas it followed neuropathy and cardiomyopathy in ATTRv. The ATTRwt patient showed muscular tracer uptake on ^99m^Tc-DPD planar scintigraphy at the time of initial diagnosis, consistent with ATTR amyloid myopathy. The ATTRv patient underwent heart transplantation because of progressive heart failure. Within the next two years, progressive myopathic symptoms and extracardiac tracer uptake on ^99m^Tc-DPD planar scintigraphy were documented, attributable to ATTR amyloid myopathy. Interstitial amyloid deposits were confirmed by muscle biopsy in both patients, with a particularly high amyloid burden in the adipose tissue. This case report highlights the frequent concomitant presence of cardiac ATTR amyloidosis and ATTR amyloid myopathy. ATTR amyloid myopathy may precede cardiac manifestation in ATTRwt or occur after heart transplantation in ATTRv. Due to the high diagnostic accuracy of ^99m^Tc-DPD scintigraphy for detecting ATTR amyloid myopathy and the emergence of novel therapeutics, it is important to increase the awareness of its presence.

## Introduction

Transthyretin (ATTR) amyloidosis is a multisystemic disorder caused by the extracellular deposition of misfolded ATTR protein in multiple organs.^1^ ATTR amyloid deposition may either be inherited (ATTR variant amyloidosis, ATTRv) or acquired (wild-type ATTR amyloidosis, ATTRwt).^2^ ATTRv amyloidosis typically presents with axonal polyneuropathy and/or cardiomyopathy, whereas additional organ manifestations and disease severity vary according to the underlying mutation.^2,3^ ATTRwt amyloidosis commonly manifests with cardiomyopathy in the elderly, although carpal tunnel syndrome, spinal cord stenosis, and tendinopathies may precede the initial manifestation of cardiomyopathy by 10-15 years.^4,5^

ATTR amyloid myopathy, which is characterized by intramuscular interstitial amyloid deposits leading to weakness in the upper and/or lower extremities may contribute significantly to morbidity.^6,7^ In contrast to immunoglobulin light chain (AL) amyloidosis, there are very few reports in the literature of amyloid myopathy in patients with ATTR amyloidosis.^6^ This is even more remarkable as Hutt et al reported extensive tracer uptake in soft tissues (mainly in the muscles) on ^99m^Tc-labeled-3,3-diphospono-1,2-propanodicarboxylic acid (^99m^Tc-DPD) scintigraphy in most patients with ATTR amyloidosis. This was particularly pronounced in ATTRwt and ATTR-Val122Ile and could be confirmed histologically as ATTR amyloid myopathy in a small subset of patients with Perugini grade 3 on ^99m^Tc-DPD scans.^7^

We herein describe two cases of ATTR amyloidosis (ATTRv and ATTRwt) with clinical, imaging, and histological evidence of ATTR amyloid myopathy, which is probably often underappreciated and underrecognized in routine clinical practice (Table 1).

**Table 1:** Clinical, laboratory, and electrophysiological characteristics of ATTR amyloid myopathy patients

	Patient 1	Patient 2
Diagnosis	ATTRv	ATTRwt
ATTR mutation	p.Val40Ile	–
Time of diagnosis	02/2015	06/2019
Age at diagnosis (years), sex	56, male	73, male
Initial ATTR manifestation	Cardiac, neuropathy	Myopathy
Myopathy (presenting symptoms)	Proximal weakness muscle cramps	Proximal weakness muscle cramps
Other symptoms at myopathy onset	Fatigue, dyspnea, leg edema, numbness, paresthesia	Absent
Weakness pattern	Proximal upper and lower extremities	Proximal lower extremity
Sensory deficits on exams	Present	Present
Creatine kinase	NI	NI
NCS/EMG		
Peripheral neuropathy	Present	Present
Motor unit potentials	Mixed pattern with high amplitudes and some short-duration potentials	Short-duration, polyphasic potentials
Fibrillation potentials in myopathic muscles	Absent	Absent

*ATTRv* transthyretin variant amyloidosis, *ATTRwt* wild-type transthyretin amyloidosis, *NCS/EMG* nerve conduction studies and electromyography, *NI* within normal limits

## Case Description

### Patient 1

#### Clinical characteristics

**Figure 1:**
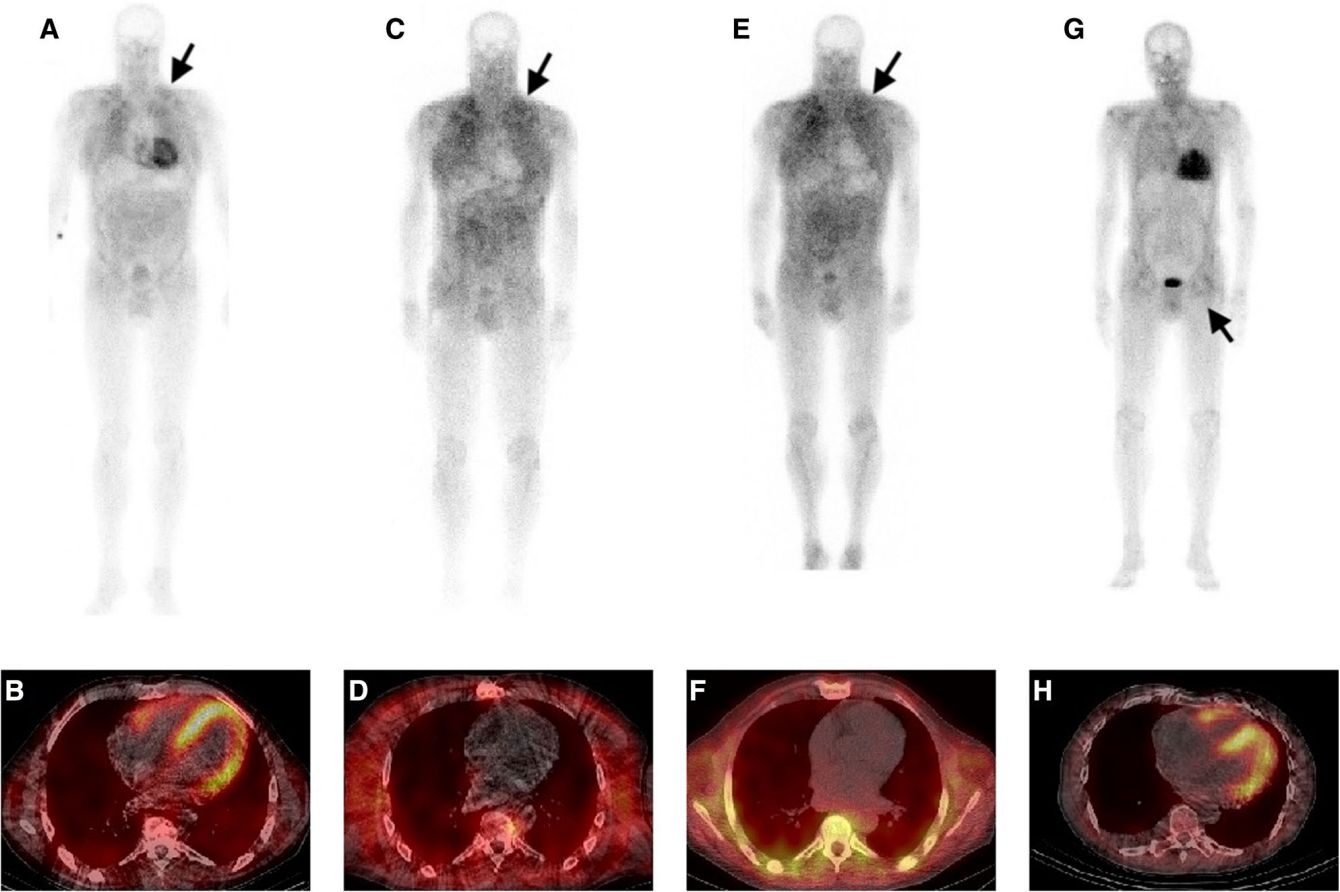
^99m^Tc-DPD whole-body planar imaging (**A**, **C**, **E**, **G**) and SPECT/CT analysis (**B**, **D**, **F**, **H**). **A** and **B** Patient 1 (ATTRv, before heart transplantation) with Grade 3 cardiac tracer uptake and moderate muscular tracer uptake both on thoracic and abdominal wall (arrow). **C** and **D** Patient 1 (ATTRv, 19 months after heart transplantation) without cardiac tracer uptake but significant muscular tracer uptake (gluteal and shoulder regions; histologically confirmed as ATTR amyloid myopathy) (arrow). **E** and **F** Patient 1 (ATTRv, 40 months after heart transplantation) without cardiac tracer uptake but visually increasing muscular tracer uptake in gluteal and shoulder regions (arrow). **G** and **H** Patient 2 (ATTRwt) with Grade 3 cardiac tracer uptake and extracardiac tracer uptake (thoracic regions and gluteal muscle; histologically confirmed as ATTR amyloid myopathy) (arrow)

**Figure 2:**
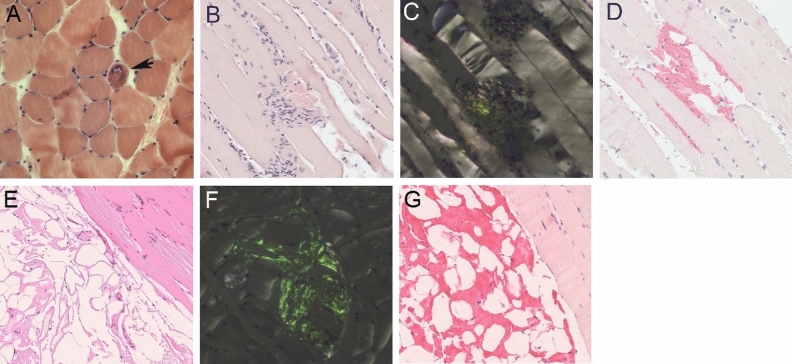
Histopathological sections of ATTR amyloid myopathy. ATTRwt, patient 2 **A** Hematoxylin & Eosin stained cross-section shows atrophic vacuolated fibers with amorphous sarcoplasmic deposits suspicious of amyloid (arrow). **B** and **C** Amyloid deposits are clearly identified in bright and polarized light on Congo red stained sections. **D** Anti-transthyretin immunostaining. ATTRv, patient 1 **E** Hematoxylin & Eosin stained tissue section shows amyloid deposits in the adipose tissue. **F** Congo red stained tissue section viewed in polarized light showing characteristic green birefringence. **G** Anti-transthyretin immunostaining

A 56-year-old man was diagnosed with cardiac ATTRv amyloidosis. Diagnosis was obtained by invasive and non-invasive methods. ^99m^Tc-DPD whole-body planar imaging (Figure 1A) and SPECT/CT analysis (Figure 1B) revealed pronounced cardiac tracer uptake (Perugini grade 3) and moderate muscular tracer uptake on thoracic and abdominal wall (Figure 1A). Endomyocardial biopsy confirmed the diagnosis of cardiac ATTR amyloidosis. Genetic testing showed a mutation in the *TTR* gene (c.118G>A; p.Val40Ile). There was no family history of amyloidosis. The patient’s medical records were remarkable for bilateral carpal tunnel syndrome ten years and decompression for lumbar vertebral stenosis two years before diagnosis of ATTRv amyloidosis. Neurological examination at the time of diagnosis of ATTRv amyloidosis in 2015 revealed fatigue, paresthesia of the feet, and loss of Achilles tendon reflexes. Dyspnea, bilateral lower leg edema, muscle cramps, mild proximal muscle weakness, upper extremity paresthesia, and numbness in the upper and lower extremities developed over the following 1.5 years. In addition to heart failure treatment, the ATTR tetramer stabilizer tafamidis was started in 2017 when this substance was first available in Austria. Three years after the initial diagnosis, the patient underwent heart transplantation due to progressive heart failure. Tafamidis, which was paused perioperatively, was restarted seven weeks thereafter. On routine follow-up 12 and 19 months after successful heart transplantation, no evidence for recurrent cardiac amyloidosis was found on endomyocardial biopsy and ^99m^Tc-DPD planar scintigraphy (Figure 1C) or SPECT/CT analysis (Figure 1D), respectively. However, in contrast to the baseline examination, ^99m^Tc-DPD planar scintigraphy revealed significant muscular tracer uptake, mainly in the shoulder and gluteal regions (Figure 1C). Muscle weakness as well as muscle cramps had increased in intensity and frequency during the following two years after heart transplantation. Biopsy of the left deltoid muscle showed significant ATTR amyloid deposition (Figure 2E-G). Due to symptom progression and confirmed intramuscular amyloid deposition, therapy was switched from the transthyretin stabilizer tafamidis to the gene silencer inotersen in 2020. This was based on the rationale that suppressing the expression of the mutant transthyretin would prevent further amyloid deposits in the muscles. On neurological follow-up in July 2021 the patient presented with normal muscle strength, while fasciculations in quadriceps and gastrocnemius muscles were frequent. Vibration perception was distally reduced (4/8 using a Rydel-Seiffer tuning fork) and Achilles tendon reflexes were absent. ^99m^Tc-DPD scintigraphy was repeated in November 2021, showing no cardiac tracer uptake on whole-body planar imaging (Figure 1E) and SPECT/CT analysis (Figure 1F). In contrast, muscular tracer uptake in the shoulder and gluteal regions was still present, visually increasing in intensity (Figure 1E). Nonetheless, the patient reported clinical improvement of muscular complaints.

#### Neurophysiological features

Quantitative sensory testing revealed thermal hypoesthesia, decreased thresholds for mechanical pain detection, and an increased wind-up ratio indicating small fiber involvement, while electroneurography showed a low-grade axonal sensory large-fiber neuropathy of lower extremities. In follow-up studies in 2018, 2020, and 2021, the neuropathy remained unchanged. Electromyography (EMG) showed minor abnormalities with a mixed pattern of mild neurogenic and myogenic changes.

#### Laboratory findings

Serum creatine kinase (CK) levels were within the normal range.

#### Muscle biopsy

Figure 2E-G and Table 2 show the myopathological findings.

**Table 2:** Myopathological findings, organ involvement, and treatment

	Patient 1	Patient 2
Diagnosis	ATTRv	ATTRwt
Muscle pathology	Present	Present
Necrotic/Regenerating fibers	None/single	None/single
Rimmed vacuoles	None	None
Nonrimmed vacuoles	Single	Single
Pathological myofiber variability	Present	Present
Congo red positive deposits	Present	Present
within muscle fibers	Absent	Scarce
within interstitial tissue	Abundant	Abundant
Tissue diagnosis		
Positive Negative	Heart, muscle	Muscle
	–	–
Organ involvement preceding myopathy	Heart, PNS	None
Organ involvement at presentation	Heart, PNS	Heart, PNS, muscle
Time from amyloidosis to myopathy onset (months)	18	0
Treatment	Tafamidis (2017-2020) Inotersen (2020 ongoing)	Tafamidis (2019 ongoing)

*ATTRv* transthyretin variant amyloidosis, *ATTRwt* wild-type transthyretin amyloidosis, *PNS* peripheral nervous system

Reticular deposits of homogeneous eosinophilic material, representing amyloid, were mainly observed in the perimysial adipose tissue. Interstitial deposition of amyloid was also found in endomysium of skeletal muscle. Interestingly, the ATTR amyloid load was greater in the surrounding adipose tissue than in skeletal muscle. Immunohistochemistry showed amyloid deposits immunoreactive for transthyretin (Figure 2E-G).

### Patient 2

#### Clinical characteristics

A 73-year-old man (ATTRwt) initially presented with bilateral lower leg edema, vertigo, numbness in the pretibial area, lower extremity muscle weakness and muscle cramps. The latter had already started 1-2 years before and was initially considered as side effect of statin use. In the absence of monoclonal proteins, diagnosis of cardiac ATTR amyloidosis was obtained by ^99m^Tc-DPD scintigraphy and cardiac magnetic resonance imaging. No pathogenic mutation could be detected and treatment with tafamidis was initiated in 2019. ^99m^Tc-DPD whole-body planar imaging (Figure 1G) and SPECT/CT analysis (Figure 1H) showed increased cardiac tracer uptake (Perugini grade 3) as well as extracardiac tracer uptake in thoracic regions and gluteal muscle (Figure 1G). Because of lower extremity muscle weakness, muscle cramps and extracardiac tracer uptake on ^99m^Tc-DPD planar scintigraphy, biopsy of the left gluteal muscle was performed 15 months (in 2020) after diagnosis of ATTRwt amyloidosis showing significant ATTR amyloid deposition (Figure 2A-D). Consequently, the patient was enrolled in a multicentric, double-blinded, randomized, placebo-controlled clinical trial in 2020, in which a next generation gene silencer is being tested in patients with cardiac ATTR amyloidosis. Therapy with tafamidis was maintained.

#### Neurophysiological features

A low-grade sensorimotor axonal polyneuropathy of lower extremities was revealed in 2019. Myopathic changes in the left gluteal muscle could be detected one year later by EMG.

#### Laboratory findings

CK levels were within the normal range.

#### Muscle biopsy

Figure 2A-D and Table 2 show the myopathological findings.

Hematoxylin and eosin staining showed slight variation of fiber size and increase of endomysial connective tissue. Necrotic fibers were not seen, although few regenerating fibers were present. Single atrophic fibers were vacuolated and contained amorphous sarcoplasmic deposits suspicious of amyloid. These amorphous deposits were clearly identified as green birefringent amyloid deposits on Congo red staining and were immunoreactive for transthyretin (Figure 2A-D).

## Discussion

In ATTRv more than 140 mutations in the *TTR* gene have been described to date, characterized by a wide phenotypic heterogeneity.^2^ The ATTR-Val40Ile variant, which was diagnosed in patient 1 is rare. Symptoms and disease progression have been investigated in the “Wagshurst study”. In this cohort including 59 individuals, the ATTR-Val40Ile variant was characterized by a high penetrance of late-onset cardiomyopathy with rapid progression of cardiac manifestation up to end-stage heart failure.^8^ A similar course was seen in patient 1, who finally underwent heart transplantation. Particularly noteworthy is the fact, that in contrast to the initial examination, ^99m^Tc-DPD planar scintigraphy 19 months after heart transplantation revealed pronounced muscular tracer uptake in the shoulder and gluteal regions in the absence of cardiac tracer uptake in the transplanted heart. At the same time the patient complained of progressive muscle weakness and muscle cramps. Biopsy from the deltoid muscle confirmed significant ATTR amyloid deposition. As there was no evidence of progression of the previously diagnosed neuropathy in the follow-up examinations, it seems likely that the patient's complaints were due to increased amyloid deposits in the peripheral muscles after heart transplantation.

One possible explanation for the development and clinical presentation of amyloid myopathy could be that ATTRv amyloid binds to pre-existing ATTRv amyloid deposits with high affinity. Therefore, after heart transplantation and release of ATTR amyloid cardiomyopathy, ATTRv amyloid may preferably deposit on pre-existing, formerly subclinical, ATTRv amyloid deposits in skeletal muscle, causing posttransplant amyloid myopathy in our patient. Similarly, it was reported that amyloid cardiomyopathy may develop after liver transplantation in ATTRv patients, due to binding of wild-type transthyretins, produced by the transplanted liver, on pre-existing ATTRv amyloid deposits in the myocardium.^9^

Hutt et al reported that most patients with cardiac ATTR amyloidosis, especially ATTRwt and ATTR-Val122Ile variant showed muscular tracer uptake on bone scintigraphy.^7^ In this study bone uptake on ^99m^Tc-DPD scintigraphy increased over the 3 h imaging period, whereas cardiac uptake and soft-tissue uptake (mainly muscle) decreased over time. This seems to be contradictory to the Perugini grading system, which is based on the reciprocal and relative decrease in bone uptake on planar imaging. The authors postulated that bone uptake particularly in patients with Perugini grade 3 on ^99m^Tc-DPD scans may be obscured by tracer uptake in overlying skeletal muscles. Extensive soft-tissue uptake may even obscure visualization of cardiac tracer uptake on planar imaging.^7^ Conversely, it is conceivable that extensive cardiac tracer uptake may obscure visualization of muscular tracer uptake. It must therefore be considered that in our patient amyloid myopathy was already present before heart transplantation but was not noticed due to this phenomenon. However, increasing muscular complaints suggest an increase in amyloid load after heart transplantation.

Despite clinical improvement of muscular complaints under gene silencing therapy, a scintigraphic follow-up examination in November 2021 (40 months after heart transplantation) still showed a marked increase in muscular tracer uptake in shoulder and gluteal regions. Whether prolonging the duration of therapy will ultimately result in regression of muscular amyloid deposits remains to be seen.

Patient 2 (ATTRwt) complained about lower extremity muscle weakness and muscle cramps, which already started 1-2 years before cardiac manifestation. This was primarily attributed to a side effect of statin therapy. However, it is more likely that these complaints were already due to the subsequently confirmed amyloid myopathy. This is in concurrence with previous reports indicating that amyloid myopathy may precede cardiac manifestation in ATTRwt.^6^

It must be noted that ATTR amyloid myopathy is often difficult to distinguish from neuropathy in routine clinical practice. This is particularly because symptoms attributed to amyloid myopathy, such as weakness and cramps, are similar to symptoms of neuropathy, CK levels are mostly within the normal range and changes in EMG are often not very pronounced. This is due to interstitial amyloid localisation, whereas muscle fiber necrosis and muscle fiber alterations, causing changes in CK levels and EMG, are rarely present. Consequently, ATTR amyloid myopathy is likely to be frequently overlooked or mistaken as neuropathy.

Histologically remarkable in this case report is the pronounced amyloid deposition in the adipose tissue in both patients, especially in patient 1 (ATTRv). Whether this makes a clinical difference from more pronounced intramuscular amyloid deposits requires further investigation. It is worth mentioning that amyloid deposits can be easily overlooked in hematoxylin and eosin staining, as seen in patient 2 (Figure 2A), underlining the need for subsequent Congo red staining. Nevertheless, ^99m^Tc-DPD scintigraphy may ultimately be the more important diagnostic screening tool for diagnosis of ATTR amyloid myopathy than biopsy in future, given the current literature.^6,7^

In a previous retrospective analysis of 57 patients with cardiac ATTR amyloidosis, Sperry et al noted that skeletal muscle uptake of ^99m^Tc-labeled-pyrophosphat (^99m^Tc-PYP) was minimal when assessed by qualitative and quantitative metrics. Thus, they conclude that the properties of ^99m^Tc-PYP may be different from ^99m^Tc-DPD in terms of non-cardiac uptake and that ^99m^Tc-PYP cannot be used to image extracardiac ATTR deposition.^10^

In summary, muscular ATTR deposits can be visualized by ^99m^Tc-DPD scintigraphy with high accuracy. As already shown in previous studies, myopathy can precede cardiac manifestation in ATTRwt amyloidosis.^6^ To date, amyloid myopathy has not been reported in the ATTR-Val40Ile variant and must be recognized as an important contributor to morbidity in these patients. In concurrence with previous reports, our findings from patient 1 (ATTRv) show that neuropathy and cardiomyopathy may precede the initial manifestation of myopathy.^6^ Whether this also applies to other patients with the ATTR-Val40Ile variant will be subject of future studies.

Conclusively, we think that ATTR amyloid myopathy is still underappreciated and underrecognized, because accurate diagnosis in the past seemed difficult to achieve in routine clinical practice. As shown in this case report, ^99m^Tc-DPD scintigraphy enables non-invasive diagnosis of ATTR amyloid myopathy with high accuracy, while invasive muscular biopsy is no longer obligatory. Due to the broad availability of ^99m^Tc-DPD scintigraphy and the emergence of novel therapeutics it is of utmost importance to increase the awareness for the frequent concomitant occurrence of ATTR amyloid cardiomyopathy and myopathy, expanding the clinical spectrum of ATTR amyloidosis.

## Data Availability

The data underlying this article are available on request from the corresponding author.

## Abbreviations

ATTR: Transthyretin
ATTRv: ATTR variant amyloidosis
ATTRwt: Wild-type ATTR amyloidosis
^99m^Tc-DPD: ^99m^Tc-labeled-3,3-diphospono-1,2-propanodicarboxylic acid
EMG: Electromyography
CK: Creatine kinase
